# Sensitive periods during the development and expression of vertebrate sexual signals: A systematic review

**DOI:** 10.1002/ece3.8203

**Published:** 2021-10-11

**Authors:** Molly T. McDermott, Rebecca J. Safran

**Affiliations:** ^1^ Department of Ecology and Evolutionary Biology University of Colorado Boulder CO USA

**Keywords:** condition dependence, divergence, indicator traits, plasticity, sexual selection, signal traits

## Abstract

Many sexually selected traits exhibit phenotypic plasticity. Despite a growing appreciation for the ecological context in which sexual selection occurs, and for the role of plasticity in shaping traits associated with local adaptation and divergence, there is an important gap in knowledge about the onset and duration of plasticity in sexual trait expression. Integrating this temporal dimension of plasticity into models of sexual selection informs our understanding of the information conveyed by sexual traits and our predictions related to trait evolution, and is critical in this time of unprecedented and rapid environmental change. We conducted a systematic review of 869 studies to ask how trait modalities (e.g., visual and chemical) relate to the onset and duration of plasticity in vertebrate sexual signals. We show that this literature is dominated by studies of coloration in birds and fish, and most studies take place during the breeding season. Where possible, we integrate results across studies to link physiology of specific trait modalities with the life stage (e.g., juvenile, breeding, or nonbreeding) during which plasticity occurs in well‐studied traits. Limitations of our review included a lack of replication in our dataset, which precluded formal analysis. We argue that the timing of trait plasticity, in addition to environmental context, is critical for determining whether and how various communication signals are associated with ecological context, because plasticity may be ongoing or occur at only one point in an individual's lifetime, and determining a fixed trajectory of trait expression. We advocate for careful consideration of the onset and duration of plasticity when analyzing how environmental variation affects sexual trait expression and associated evolutionary outcomes.

## Graphical abstract

Despite a growing appreciation for the ecological context in which sexual selection occurs, there is an important gap in knowledge about the onset and duration of plasticity in sexual trait expression. We conducted a systematic review of 869 studies to ask how trait modalities (e.g., visual and chemical) relate to the onset and duration of plasticity in vertebrate sexual signals. We argue that the timing of trait plasticity, in addition to environmental context, is critical for determining whether and how various communication signals are associated with ecological context, because plasticity may be ongoing or occur at only one point in an individual's lifetime, and determining a fixed trajectory of trait expression.

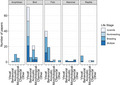

## 1 INTRODUCTION

Sexually selected phenotypes are some of the most notable features of wild animals and have fascinated biologists since Darwin proposed their function in reproductive behavior (Darwin, 1896). A key question in sexual selection research is why certain traits, but not others, become the targets of sexual selection. Several models propose that traits favored by sexual selection are indicative of good genes and health in a given ecological context (“indicator trait models,” Table 1; Andersson & Simmons, 2006; Cornwallis & Uller, 2010) and that the evolution of multiple signals may be selected for because each trait conveys unique information about the signaling animal (“multiple message hypothesis”; Møller & Pomiankowski, 1993). Indeed, decades of research has identified (1) specific examples of plasticity in sexual traits, and (2) the types of environmental factors that influence expression of sexual traits. For example, parasite load influences male color of house finches (*Haemorhous mexicanus)* (Hill & Farmer, 2005; Thompson et al., 1997), and diet quality influences attractive chemosignals of Iberian mountain lizards *Iberolacerta monticola* (Martín & López, 2006, 2015).

**TABLE 1:** Examples of indicator trait models and the critical life stage that influences either or both trait development and expression in this model

Model	Predictions	Critical life stage
Indicator trait models		
Handicap principle^a^	Sexual traits are costly; therefore, only some individuals can afford to develop exaggerated traits	Any
Hamilton‐Zuk^b^	Sexual traits contain information about an individual's ability to resist costly parasites	Any
Good parent^c^, ^d^	Sexual traits advertise nonheritable variation in parental care—healthy males will be better caregivers of offspring	Breeding or nonbreeding
Developmental stress^e^	Song learning and other neurological traits are sensitive to stress in early development and are informative about traits developed simultaneously	Juvenile

^a^ Zahavi (1975).

^b^ Hamilton and Zuk (1982).

^c^ Hoelzer (1989).

^d^ Wolf et al. (1997).

^e^ Nowicki et al. (1998).

Research on sexually selected traits has shown that the degree of trait exaggeration is associated with environmental context (Martín & López, 2015; Scheuber et al., 2003a; Thompson et al., 1997). However, we still lack a clear understanding about the *timing* of trait sensitivity to the environment throughout an individual's life (e.g., “static” vs. “dynamic” signals; *sensu* Hebets & Papaj, 2005). Research in human learning and mammal behavior has, for decades, recognized the existence of “sensitive periods” in development during which environmental influences (esp. social interactions and neurotoxins) have a profound effect on developmental trajectories (Illingworth & Lister, 1964; Rice & Barone, 2000). Many neural traits show patterns of developmental plasticity (Bateson et al., 2004; Stamps, 2016; West‐Eberhard, 2005), which we define here as traits that are most sensitive to environmental variation in early life. Studies of insect metamorphoses and migration show that plasticity can be induced by seasonal changes in the environment, that is, seasonal plasticity (Nylin, 1992; Nylin et al., 1989). Behavioral ecologists recognize how moment‐to‐moment changes in the environment alter an animal's behavior, that is, continuous or contextual plasticity (Stamps, 2016). These types of plasticity differ in onset and duration of a sensitive period in trait expression (Table 2).

**TABLE 2:** Conceptual outline of trait–environment correlations at different life stages and examples of types of traits might display these patterns and the implications for mate choice and population divergence. These simplified examples are not exhaustive, but are intended to outline patterns of plasticity that are likely common in sexual traits

Evidence of plasticity			Pattern	Example trait types	Implications for mate choice	Implications for population divergence
Juvenile	Breeding	Nonbreeding (if applicable)				
No	No	No	No environmental sensitivity at any life stage	Some traits are not plastic, and for those that are, some environmental variables will have no effect	If trait is not plastic, there is a strong genetic basis and trait signals indirect (genetic) benefits to offspring	If gene flow is reduced, these traits could diverge rapidly
Yes	No	No	Sensitive to environmental variable during juvenile period, but not afterward (developmental plasticity)	Neural traits underlying song learning and some behavioral patterns^a^	Delays between sensitive period and trait expression may erode real‐time signal reliability; however, trait may be informative about quality of natal and early‐life environment, including parental care	If preferences are formed by imprinting or traits are learned through tutoring, trait divergence can occur and be maintained even in the presence of gene flow
No	Yes	No	Sensitive to environmental variable during breeding season, but not at other times (seasonal plasticity)	Traits that depend on short‐term metabolic investment and are only expressed during the breeding season/after sexual maturity, such as acoustic display rates in frogs^b^	Trait is informative about recent health, useful for mate choice contexts in which direct benefits are important	In the case of a migratory divide where populations share a breeding location, may erode divergence
No	No	Yes	Sensitive to environmental variable during nonbreeding season, but not at other times (seasonal plasticity)	Signals in tissues grown seasonally during the nonbreeding season, such as bird plumage^c^	Delays between sensitive period and trait expression may erode signal reliability; however, trait may be informative about resources acquired during the nonbreeding growth period (e.g., nutritional resources)	In the case of a migratory divide where populations share a breeding location, may contribute to divergence
Yes	Yes	Yes	Continued sensitivity to environmental variable across life stages (contextual or continuous plasticity)	Traits that require ongoing or short‐term metabolic investment but capacity for trait expression is shaped continuously by the environment, starting with early‐life conditions, such as chemical signaling in lizards^d^	Trait integrates information about health over life span. May contribute to signal unreliability or may be informative about multiple life stages simultaneously	Novel environment may rapidly affect the expression or honesty of a trait, potentially changing selection on preferences and contributing to divergence

^a^ MacDougall‐Shackleton and Spencer (2012).

^b^ Cunnington and Fahrig (2010).

^c^ Saino et al. (2004).

^d^ Martín and López (2015).

Understanding the physiological mechanisms underlying sensitive periods more generally, and the implications for evolution, is a topic of intense recent interest and an explosion of research (Boyce et al., 2020; Reh et al., 2020; Sanz et al., 2020). While theories of multiple signal evolution have acknowledged the roles of both timing of sensitive periods and type of environmental variable (e.g., parasites vs. nutrition) as contributing to the information conveyed by signal traits (Candolin, 2003; Møller & Pomiankowski, 1993), empirical work on sexually selected traits is usually designed to detect types of influential environmental variables (Cornuau et al., 2014; Hill et al., 2009; Rahman et al., 2013) rather than timing (but see Scheuber et al., 2003a, 2003b for excellent counterexamples). Researchers examining the physiological basis of sexual signals likely choose to study their animals during a stage they believe to be sensitive to a particular environmental variable based on their prior knowledge of the system, but this is rarely if ever made explicit.

In sexual selection research, environment–trait linkages are frequently described as condition dependence (Andersson, 1986; Hill, 2011; Rowe & Houle, 1996; Warren et al., 2013). Although there is much debate about how to define and measure condition (Wilson & Nussey, 2010), condition dependence is increasingly conceptualized as a special case of phenotypic plasticity in which trait expression is exaggerated when an individual experiences favorable environments (Cornwallis & Uller, 2010; Fox et al., 2019; Price, 2006), for example, bright coloration in an individual with access to a high‐quality diet or no parasitic infections (Hill & Farmer, 2005; Ruell et al., 2013). Accordingly, to adopt consistently defined terminology, we refer to the environmental influences on trait development and expression as the extent to which a trait exhibits plasticity.

In this review, we seek to integrate an explicit consideration of sensitive periods in sexual trait expression with decades of research focused on the evolution of sexually selected traits. Considering the temporal dimension of plasticity in sexually selected traits may help to integrate results that at first seem contradictory but instead point to variation in the sensitivity to the environment during trait development or expression. For example, food quantity seems to affect song frequency in *Taeniopygia guttata* during the juvenile stage (Woodgate et al., 2014) but not during the adult stage (Ritschard & Brumm, 2012). If one were to study the sensitivity of song frequency to food quantity during either season, one would not get a complete picture of how this sexual signal is influenced by the environment. Here, we suggest that the timing of environmental influence on sexual trait development (i.e., the existence of sensitive periods) is an understudied yet critical component of understanding what information is conveyed by sexual traits and how they evolve (Box 1).

BOX 1 **Implications for mate choice**

*Developmental plasticity*. Social factors influence song type in zebra finches during early development, but once learned, song type is fixed for the duration of the individual's lifetime (Holveck et al., 2008). Therefore, song type may provide information about the social environment, but only the social environment during early life. The developmental stress hypothesis suggests that sexual traits learned in early life, such as song, may provide overt clues about less obvious neural traits developed at a similar developmental timeframe (Buchanan, 2011). Scenarios where there is a delay between the environmentally sensitive period and changes in trait expression have been highlighted as a mechanism by which plasticity in sexual traits may erode signal reliability (Ingleby et al., 2010), but they may also be informative, as when traits are correlated with the quality of parental care received by an individual and thus likely to be provided by that individual (Hoelzer, 1989; Wolf et al., 1997).

*Seasonal plasticity*. Traits such as antlers and plumage are molted and regrown seasonally, and it is during the seasonal growth period that environmental influences such as parasite load and nutrition affect trait expression. This implies that a trait such as plumage color may provide information about parasite load, but only at the time of molt. Knowledge of the study organism plays a key role in identifying this period of heightened sensitivity. For example, in birds, feather coloration is grown during a molt providing a clear biological time point during which pigment deposition occurs and is thus likely to be more sensitive to environmental influences. Mates can assess seasonally grown traits for information about an individual's health during the seasonal growth period, which may be particularly important if territories or other resources are acquired during the seasonal growth period. Similar to developmental plasticity (see above), delays between the sensitive period and trait expression may erode signal reliability (Ingleby et al., 2010). Seasonally grown traits may also be evolutionarily constrained and not ideal signals of mate quality. Bird feathers, for example, must be molted to maintain flight performance, but, once regrown, are mostly dead tissues and pigment deposition ceases.

*Continuous plasticity*. If a trait is continuously sensitive (plastic) throughout its lifetime, that trait may provide a reliable real‐time readout of an individual's ability to cope in a particular environment. For example, chemical signals can change with relatively short‐term changes in diet or stress (e.g., Martín & López, 2006). There is little delay between changes in the environment and changes in trait expression. Thus, potential mates are able to assess the recent or real‐time health of the signaling animal. This may be particularly relevant when direct benefits or territory quality are critical for ensuring the survival or reproductive success of the mate or offspring.

We systematically reviewed literature on sexually selected traits across vertebrate taxa to discern which life stages have been studied for influence on the development of sexual traits (i.e., how broadly are sensitive periods considered?) and whether different trait modalities (e.g., visual and chemical) have predictable patterns in the onset and duration of sensitive periods. Our review yielded the following insights: (1) Timing of trait sensitivity to environmental influences has rarely been emphasized in sexual selection research, (2) a temporal understanding of plasticity can integrate results of different studies, which provide seemingly conflicting evidence about trait–environment associations, and (3) the onset and duration of sensitive periods in sexual signals has important implications for sexual trait expression and evolution.

## 2 METHODS

We compiled relevant papers by searching Web of Science on August 27, 2020, with the keywords “taxon, trait, sexual selection, and condition‐depend*” and on March 30, 2021, with the keywords “taxon, trait, sexual selection, and plasticity” where taxon was one of five vertebrate groups: amphibian, bird, fish, mammal, or reptile; and trait was one of five trait modalities: acoustic, behavioral, chemical, morphological, or visual. Using all possible taxon–trait keyword combinations resulted in 50 unique searches. Due to the diverse terminology and study systems used by researchers studying sexual selection, our search is not exhaustive. Rather, we intended to capture a representative sample of vertebrate research in sexually selected traits over the last few decades. We chose to use the term “condition dependence” as well as “plasticity” in our searches as this is the most widely used terminology across years and taxonomic groups to study trait plasticity in the context of sexual selection.

**FIGURE 1:**
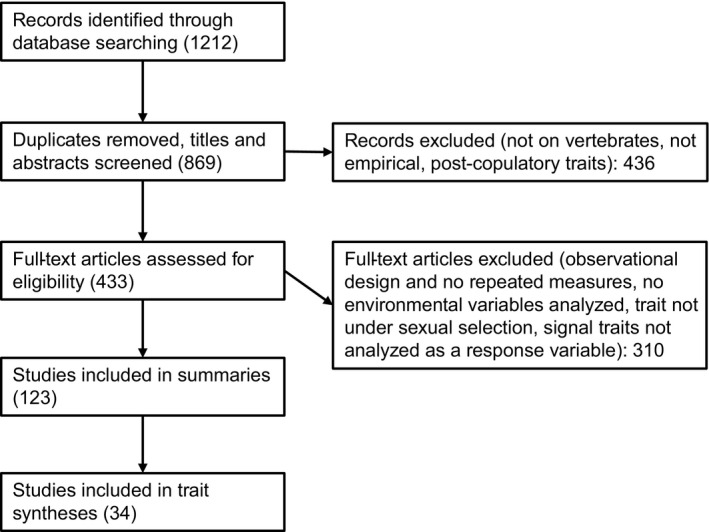
Flowchart showing database results and filtering at each stage of our systematic search

We systematically screened all titles and abstracts and, if necessary, methods and results to exclude papers that: were on invertebrates, only considered traits other than sexually selected pre‐mating traits (e.g., sperm or a naturally selected trait), did not contain data at the level of the individual, were not empirical (e.g., reviews or theoretical studies), or did not manipulate or measure an environmental variable (Figure 1). We chose to focus on vertebrate research here to fill a gap in the literature about sexual selection and plasticity. To our knowledge, no such review exists for vertebrates; however, similar topics have been touched on in reviews focused on invertebrates (Hunt & Hosken, 2014; Ingleby et al., 2010). In addition, we hoped to make some generalizations about physiological mechanisms behind trait expression at particular life stages, which would not be possible when reviewing vertebrates and invertebrates together. We further wished to focus on the information potential mates can obtain from signal traits, and therefore, we did not include papers on post‐copulatory traits or those used primarily in male–male aggression, although these are certainly interesting and exciting subjects of study (for a nice review of behavioral plasticity in pre‐ and post‐copulatory traits, see Bretman et al., 2011). We focused on papers that had manipulated an environmental variable of interest (hereafter “experimental studies”) or those that used a repeated‐measure design to track individual change over time in response to environmental change (hereafter “longitudinal studies”). Observational studies without repeated measures (i.e., individuals are measured at only a single point in time) cannot detect plasticity, since any correlations between trait and environment could be due to genetic differences, plasticity, or both.

We thoroughly read and scored all selected papers with particular attention to life stage, study design (experimental, longitudinal, or both), types of environmental variables, types of traits, and the quality of evidence that the trait of interest is sexually selected. For a detailed list of scoring criteria and notes, see Table S2. We ensured consistency by having both observers [MTM and RJS] score the same set of five papers and refined our definitions in light of any discrepancies. We repeated this process for four sets of five papers until we had complete agreement in scoring among observers. Afterward, papers were scored independently. Any papers where we felt uncertain about categorization were scored together.

To answer our driving questions about the state of the literature in the context of the timing of sexual trait plasticity, we summarized the distribution of papers among years, taxa, traits, environmental variables, experimental designs, and life stages. We also summarized the number of traits or environmental variables studied by these papers. To gain a deeper understanding of the findings of this body of literature, we identified traits in our database that (1) were studied across multiple life stages, (2) had good evidence for being sexually selected, that is, were correlated with mating or reproductive success, and (3) were measured in a relatively consistent way such that a synthesis across studies was possible. For each of the papers on these well‐studied traits, we scored the trait–environment association as positive, negative, or not significant. We then synthesized the results to gain insight about the onset and duration of sensitive periods in that trait's development. Unfortunately, a meta‐analysis of sensitive periods across trait modalities was not possible due to insufficient replication across life stages within taxa and the diversity of analysis techniques (e.g., PCA, model selection, and generalized linear mixed models) used to study the effects of environment on trait expression.

## 3 RESULTS

**FIGURE 2:**
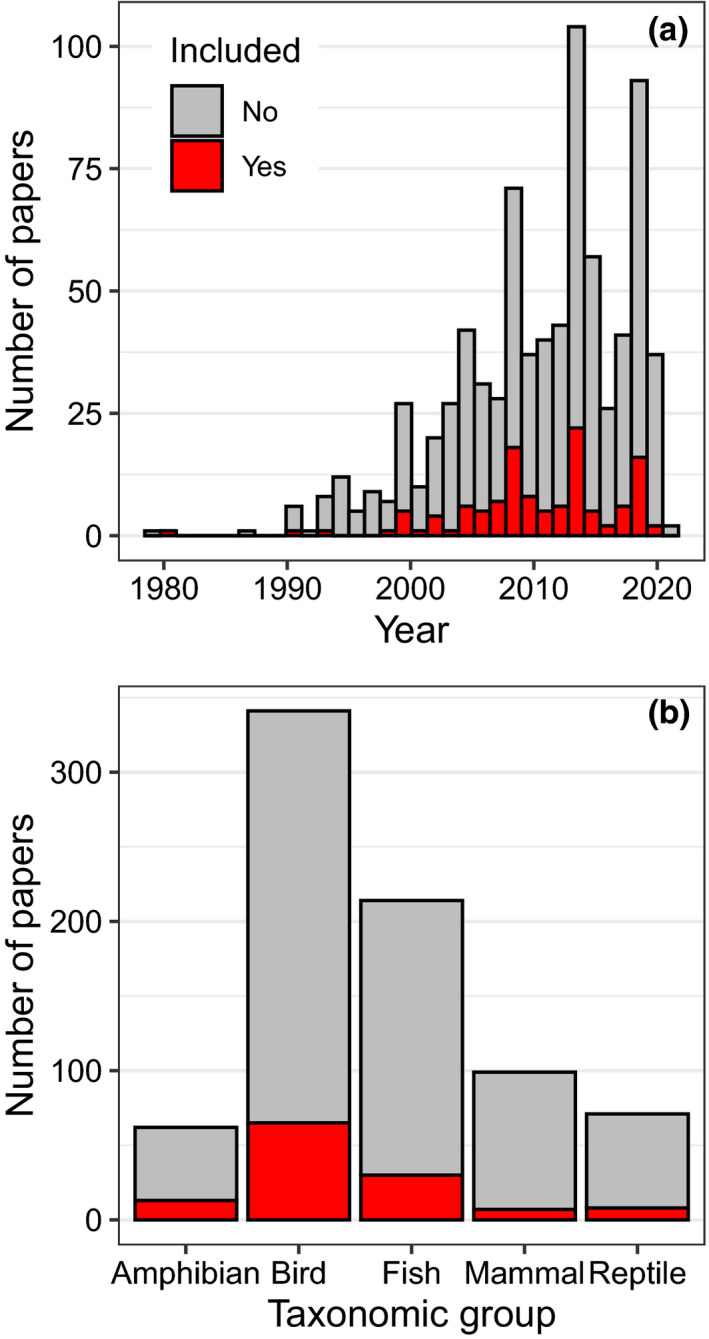
Summary of search results and selected papers by (a) taxonomic group and (b) year. Papers that met criteria for inclusion are shown in red

Our searches returned 869 unique papers (Figure 1; Table S1). Of these, 123 (14%) met our criteria for inclusion in the summaries below. Selected papers were representative of the overall search results in years published (Figure 2a) and taxonomic group studied (Figure 2b). Birds are by far the most well‐studied group in this literature, followed by fish. The most common reason a paper was excluded was due to the use of a purely observational design where data were collected at one time point, because we focused on experimental and longitudinal studies that allow researchers to detect trait plasticity.

**FIGURE 3:**
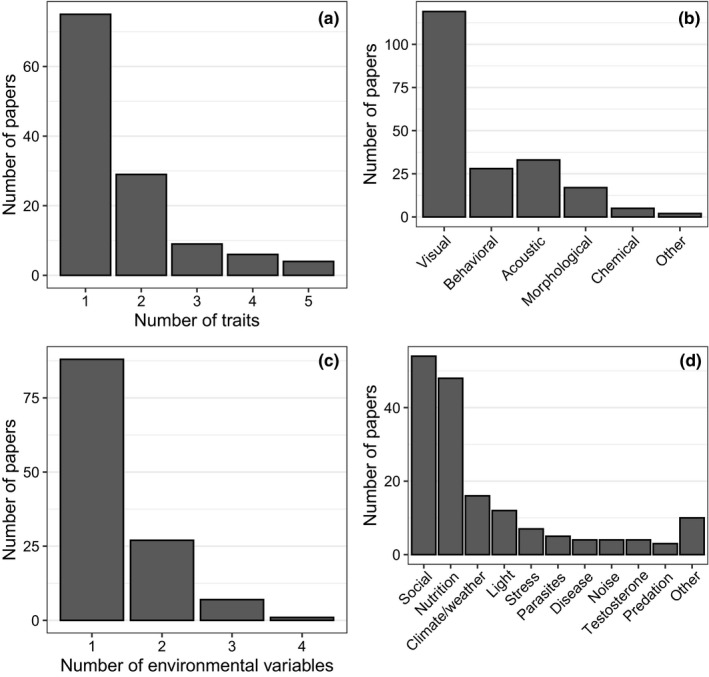
Summary of (a) number of traits, (b) trait modalities, (c) number of environmental variables, and (d) types of environmental variables considered in 123 papers that met our selection criteria. If a paper studied multiple types of traits or environmental variables, these were scored separately so the totals in (b) and (d) add to more than 123. “Other” environmental variables include water quality, preening effort, tail removal, previous reproductive effort, and administration of sex hormones

**FIGURE 4:**
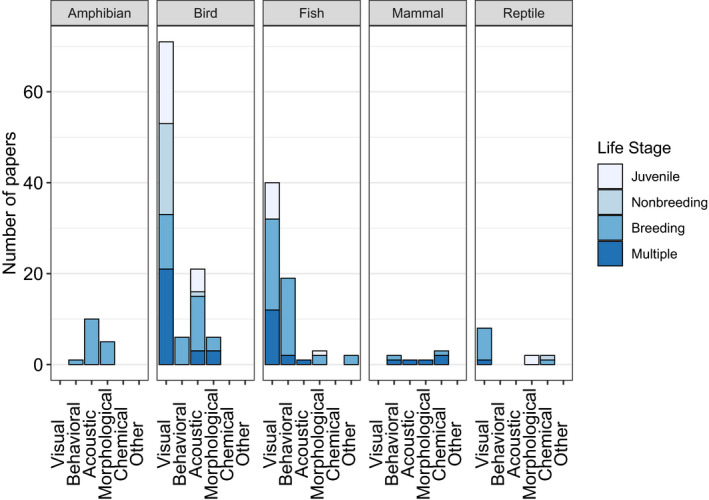
Summary of life stages studied by trait modality and taxonomic group. If a paper studied multiple trait modalities, these were scored separately so the totals add to more than 123

Visual signal traits were the most commonly studied and chemical traits the least‐well represented among selected papers (Figure 3a). Nutritional and social aspects of the environment were most frequently studied for effects on signal traits (Figure 3b). Most selected papers studied one signal trait (Figure 3c). A similar pattern emerged when considering environmental variables; most papers studied just one environmental variable (Figure 3d). Typically, each combination of taxonomic group and trait modality had been analyzed in one or two life stages in our collection of studies (Figure 4). Acoustic and visual traits in birds and visual, behavioral, and morphological traits in fish were the only traits for which all relevant life stages were studied (for many fish, there is no nonbreeding season), and thus, temporal patterns of plasticity (Table 2) could potentially be assessed only in these taxa.

All of our selected papers framed the trait(s) in question as a sexual signal, reproductive trait, or similar. However, the evidence given for sexual selection (i.e., a correlation with mate choice and/or reproductive success) was often weak or nonexistent. Over a third of the traits in selected papers were not clearly linked with mate choice or reproductive success (87/203 traits or 43%, total is greater than the number of selected papers because some papers studied multiple traits). In these cases, the authors either did not provide citations for statements describing a trait as sexually selected or provided indirect evidence (e.g., the trait is sexually dimorphic, linked with territory quality, or more common among dominant individuals). About half of the papers did cite evidence of the trait being linked with mate choice and/or reproductive success (99/203 traits or 49%), and a few exceptional papers directly tested the link with mate choice and/or reproductive success, as well as trait plasticity (17/203 or 8%; see Head et al., 2017; Martín & López, 2006 for good examples).

### 3.1 Trait syntheses

The most well‐represented species among selected papers were guppies (*Poecilia reticulata*, 14 papers), zebra finches (*Taeniopygia guttata*, 10 papers), three‐spined sticklebacks (*Gasterosteus aculeatus*, seven papers), house sparrows (*Passer domesticus*, six papers), and great tits (*Parus major*, five papers). Together, these species represented over a third of the papers in our dataset. We identified four traits that met our criteria for further synthesis: song characteristics in zebra finches, sigmoid displays in guppies, orange coloration in guppies, and black badge size in house sparrows (see Methods; in brief, traits had to be (1) studied in multiple life stages, (2) sexually selected, and (3) measured consistently across studies). Below, we report what environment–trait associations have been found at different life stages and in response to different environmental variables for these traits. Furthermore, we integrate the results of different studies to highlight potential patterns of developmental, seasonal, or continuous plasticity in sexual trait development.

#### 3.1.1 Orange spot size in guppies

**FIGURE 5:**
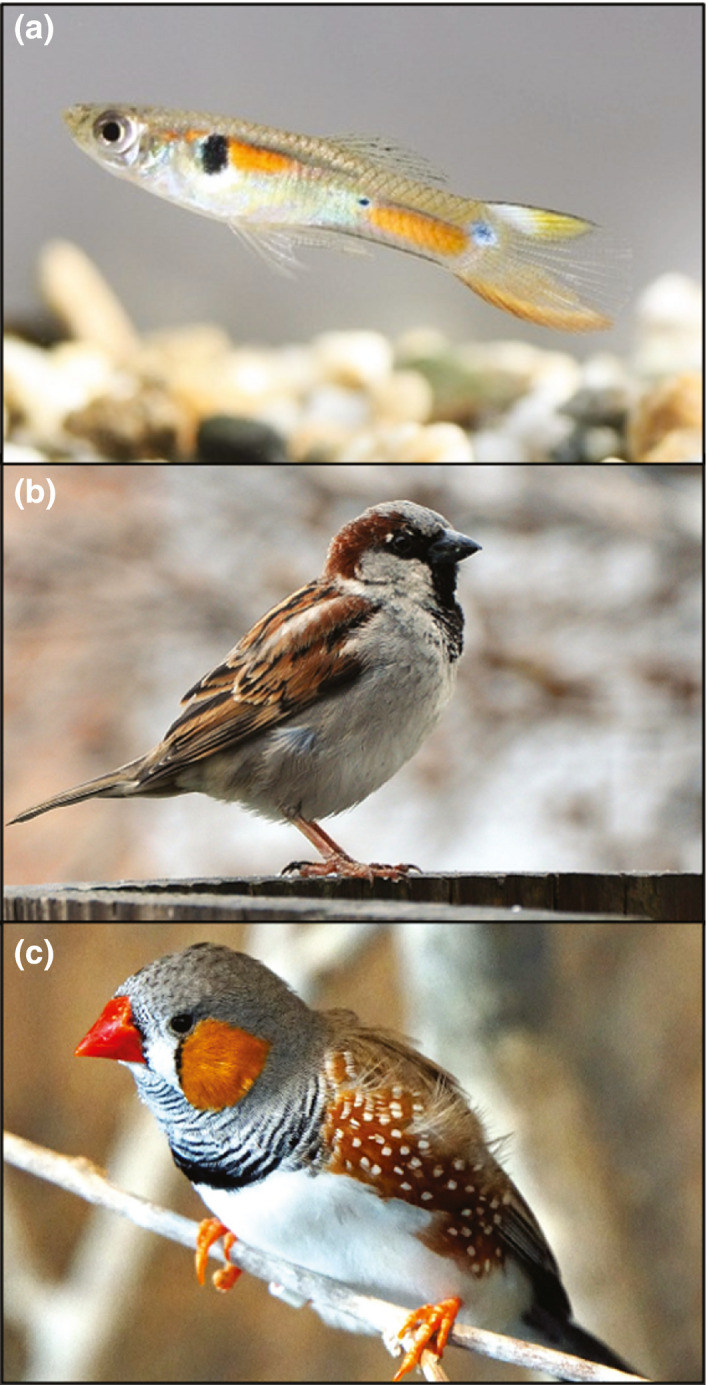
Animals that are the subject of our review's trait syntheses shown are (a) a male guppy (*Poecilia reticulata*), photograph by Clelia Gasparini under CC‐BY 2.5; (b) a male house sparrow (*Passer domesticus*), photograph by Joe Ravi under CC‐BY‐SA 3.0; and (c) a male zebra finch (*Taeniopygia guttata*), photograph by Dennis Jarvis under CC‐BY‐SA 3.0

Guppies (Figure 5a) are live‐bearing freshwater fish with a polyandrous mating system that has become a model system in sexual selection research. Male guppies display orange, yellow, white, black, and iridescent coloration in highly variable and individually unique patterns (Houde, 1997). The guppy's orange spots are carotenoid‐based, a pigment known to be influenced by nutrition in many species as it cannot be synthesized by animals and must be acquired through the diet (Goodwin, 1984; Schiedt, 1989). The extent of orange coloration influences mate choice, with females preferring to mate with males who display more conspicuous orange coloration (Endler, 1984; Kodric‐Brown, 1985). For details on how genotype‐by‐environment interactions shape sexual selection in this species, see Kolluru (2014).

Our selected papers included seven studies that tested the effects of environmental variables on orange spot size (Table 3). Environmental manipulations took place before and after sexual maturity (juvenile and breeding life stages, respectively). These studies did not all test the same environmental variables, but there is considerable overlap: Five studies manipulated some aspect of nutrition, likely because of the known physiological link between carotenoid pigmentation and diet. Four of these studies manipulated the quantity of food experimental fish received (Devigili et al., 2013; Evans et al., 2015; Rahman et al., 2013; Ruell et al., 2013), and two manipulated the carotenoid content of fish diets (Grether, 2000; Rahman et al., 2013). The two remaining studies (Miller & Brooks, 2005; and Magris et al., 2018) tested the effects of the social environment (namely, the number and continued presence of females) but found no effect on orange coloration in either the juvenile or breeding stage.

**TABLE 3:** Summary of findings from studies on traits that (1) were studied across multiple life stages, (2) had good evidence for being sexually selected, and (3) were measured in a relatively consistent way such that comparison across studies was possible

Scientific Name, Trait	Paper	Sex	Life stage(s)	Evidence for plasticity?	Environmental variable type	Environmental variable	Environment–trait correlation	Treatment duration
*Passer domesticus*, black bib size	Dupont et al. (2019)	M	Juvenile	Yes	Stress	Corticosterone in diet	(‐)	1 year
	Gonzalez et al. (1999)	B	Juvenile	No	Nutrition	High‐ or low‐protein diet	ns	12 weeks
		B	Juvenile	No	Disease	Immune functioning	ns	12 weeks
	Griffith (2000)	M	Carryover (breeding to breeding)	Yes	Other	Brood size previous year	(‐)	1 year
	Griffith et al. (1999a)	M	Juvenile	Yes	Social	Foster father badge size	(+)	4–5 months
		M	Juvenile	Yes	Climate/weather	Date of hatch	(‐)	4–5 months
		M	Juvenile	No	Nutrition	Parental feeding rate	ns	4–5 months
	McGraw et al. (2003)	M	Nonbreeding + Breeding	Yes	Social	Aggression	(+)	8 months
	McGraw et al. (2002)	M	Nonbreeding	No	Nutrition	Food quantity	ns	3 months
*Taeniopygia guttata*, syllable repertoire	Gil et al. (2006)	M	Juvenile	No	Social	Brood size	ns	3–6 months
		M	Juvenile	Yes	Social	Tutor identity	(+)	3–6 months
		M	Juvenile	Yes	Social	Number of males in tutoring group	(+)	3–6 months
	Greene et al. (2018)	M	Juvenile + Breeding^a^	No	Nutrition	Mercury in food or not	ns	~1 year
	Holveck et al. (2008)	M	Juvenile	No	Social	Brood size	ns	140 days
	Noguera et al. (2017)	M	Juvenile	Yes	Nutrition	Micronutrient levels	(+)	5–8 months
	Ritschard and Brumm (2012)	M	Breeding	No	Nutrition	Food quantity	ns	9–15 weeks
	Woodgate et al. (2014)	M	Juvenile	Yes	Nutrition	Food quantity	(+)	~165 days
*Poecilia reticulata*, Orange spot size	Devigili et al. (2013)	M	Breeding	Yes	Nutrition	Food quantity	(+)	41 days
	Evans et al. (2015)	M	Breeding	Yes	Nutrition	Food quantity	(+)	4 months
	Grether (2000)	M	Juvenile	No	Nutrition	Carotenoid supplementation	ns	Unknown
	Magris et al. (2018)	M	Breeding	No	Social	Constant vs. Changing number of females	ns	2, 3, and 6 months
	Miller and Brooks (2005)	M	Juvenile + Breeding	No	Social	Access to females	ns	100 days
	Rahman et al. (2013)	M	Breeding	Yes	Nutrition	Food quantity	(+)	4 months
		M	Breeding	No	Nutrition	Carotenoid supplementation	ns	4 months
	Ruell et al. (2013)	M	Juvenile	Yes	Predation	Predation risk	(‐)	78 days
		M	Juvenile	Yes	Nutrition	Food quantity	(+)	78 days
*Poecilia reticulata*, sigmoid display rate	Chapman et al. (2009)	M	Juvenile	No^b^	Light	Light level	ns^b^	18–20 weeks
		M	Breeding	No^b^	Light	Light level	ns^b^	1 day
	Devigili et al. (2013)	M	Breeding	Yes	Nutrition	Food quantity	(+)	41 days
	Ehlman et al. (2018)	M	Breeding	Yes	Light	Water turbidity	(+)	30 min
	Evans et al. (2015)	M	Breeding	Yes	Nutrition	Food quantity	(+)	4 months
	Farr (1980)	M	Breeding	No	Social	Female receptivity	ns	3 weeks
	Jordan and Brooks (2012)	M	Breeding	No	Social	Females’ size variance	ns	1 week
		M	Breeding	Yes	Social	Females presented simultaneously or sequentially	(+) sequential	1 week
	Kelley et al. (2013)	M	Breeding	No	Light	Light level	ns	<10 min, 1 week between treatments
	Kolluru et al. (2015)	M	Breeding	Yes	Light	Light level	(‐)	15 min
	Miller and Brooks (2005)	M	Juvenile + Breeding	Yes	Social	Access to females (none, courtship, mating)	(+) courtship	100 days
	Rahman et al. (2013)	M	Breeding	Yes	Nutrition	Food quantity	(+)	4 months
		M	Breeding	No	Nutrition	Carotenoid supplementation	ns	4 months
	Reynolds (1993)	M	Breeding	No^c^	Light	Light level	ns^c^	15 min

^a^ Subjects were also exposed to mercury in ovo.

^b^ Significant interaction between rearing environment and display environment.

^c^ Significant interaction with size.

Considering life stage (breeding or juvenile) helps explain when results of these studies agree or disagree with one another. The key is that inferences about trait plasticity from each study are limited to the time point in which the trait was analyzed and the environmental variable under consideration. When dietary manipulations took place during the juvenile stage, no association was found between carotenoid supplementation (Grether, 2000) or food quantity and orange spot size at maturity (Ruell et al., 2013). In contrast, when dietary manipulations took place after reaching sexual maturity (breeding), food quantity was positively associated with orange spot size (Devigili et al., 2013; Evans et al., 2015; Rahman et al., 2013). This pattern indicates orange spot size may reflect environmental conditions experienced by male guppies at sexual maturity, but is not indicative of their early developmental environment, in accordance with a pattern of seasonal (breeding) plasticity (Table 2). Comparing the effects of nutritional manipulations with social manipulations highlights the point that a lack of evidence for trait plasticity is not evidence that a trait is not plastic. Rather, a trait may be sensitive to environmental variables and/or at life stages other than those being studied.

#### 3.1.2 Sigmoid displays of guppies

In addition to coloration, male guppies use a ritualized courtship behavior termed the “sigmoid display.” This display is thought to show off a male's color patterns and display rates are linked with increased reproductive success (Houde, 1997).

Our selected papers included 11 papers that analyzed plasticity of sigmoid display rates at different life stages (Table 3), although most of these studies manipulated the environment after sexual maturity (breeding). These studies manipulated light environment (light intensity or water turbidity, five studies), social conditions (type, number, size, and presence of females, three studies), and nutrition (food quantity or carotenoid supplementation, three studies).

These studies provide mixed evidence for the effect of light levels during courtship (breeding) on display rates. Where some studies found no evidence of a correlation (Chapman et al., 2009; Kelley et al., 2013; Reynolds, 1993), others found that male guppies tended to display more in low light conditions (Kolluru et al., 2015) or high turbidity (Ehlman et al., 2018). Importantly, there is evidence that individual males may respond to lighting conditions differently depending on their rearing environment or size, as evidenced by a significant interaction with display rates (Chapman et al., 2009; Reynolds, 1993), and this individual variation may help to explain the apparent discrepancy between studies with similar methods.

The four studies testing social conditions did not all find evidence for a correlation with display rates, but these studies manipulated different aspects of social conditions. Neither female receptivity (Farr, 1980) nor variance in female size (Jordan & Brooks, 2012) was linked with display rates. The order and number of females present over a week did influence display rates, with sequential presentation of females resulting in higher display rates of male guppies (Jordan & Brooks, 2012). The one study that manipulated juvenile social conditions found that males who were allowed to court, but not mate with, females displayed more often than males housed separately from females or those who were allowed to mate with females (Miller & Brooks, 2005). This study manipulated social conditions continuously from before to after sexual maturity (juvenile and breeding), and therefore, it is not possible to determine whether display rates were sensitive to this manipulation before sexual maturity, afterward, or both.

Nutrition was only studied at one life stage (breeding), and across studies, food quantity was associated with increased display rates. Consistent with previous work that has established the continuous plasticity of behavioral traits (Table 2; Stamps, 2016), there is evidence that even very short‐term changes in lighting, social conditions, and nutrition can affect the display rates of male guppies; however, the evidence is somewhat mixed (Table 3). On the contrary, there is also evidence that early‐life conditions can interact with current conditions to influence individual display rates, pointing to the potential importance of juvenile environment in shaping behavioral patterns of guppies.

#### 3.1.3 Chest badge size in house sparrows

House sparrows (Figure 5b) are an extremely common, widespread songbird that have been studied extensively in aviaries and natural settings. They are sexually dimorphic, with males displaying a prominent black patch of feathers (badge or bib) on their throat and chest. This patch is melanin‐based, and the size of the patch has been associated with both inter‐ and intrasexual selection (Griffith et al., 1999b; Møller, 1988), although more recent meta‐analyses point to a lack of evidence for sexual selection (Nakagawa et al., 2007; Sánchez‐Tójar et al., 2018).

Included in our review were six studies that analyzed the environmental sensitivity of badge size at different life stages (Table 3): Two of these studies were conducted on juvenile birds (Gonzalez et al., 1999; Griffith et al., 1999a) and one on nonbreeding birds (McGraw et al., 2002). None of these three studies found an effect of nutritional conditions on badge size.

In contrast, badge size increased with aggressive social conditions during molt (McGraw et al., 2003) and decreased with greater breeding effort in the prior breeding season (Griffith, 2000). Therefore, badge size is plastic during molt (nonbreeding) but is likely more sensitive to social environment and prior reproductive effort than to nutritional conditions. Taken together, these results are consistent with a pattern of seasonal plasticity during molt (Table 2), although it may be plastic at other time points as well.

#### 3.1.4 Syllable repertoire in zebra finches

Zebra finches (Figure 5c) are a small, socially monogamous songbird that have been used extensively in laboratory studies, mainly in studies of neural development. Zebra finches sing a complex, rhythmic song that is a multicomponent signal. High syllable repertoire is among the song traits preferred by females (for a detailed review of song function and sexual selection in zebra finches, see Riebel, 2009). Included in our review were six studies that analyzed syllable repertoire (also referred to as number of unique syllables or elements; Table 3).

Once again, considering life stage helps when interpreting these studies together. Four studies manipulated nutritional conditions, but only during the juvenile stage is there evidence for effects on syllable repertoire (Noguera et al., 2017; Woodgate et al., 2014). Manipulations of food quantity during the breeding season did not affect syllable repertoire (Ritschard & Brumm, 2012), and exposure to mercury did not affect syllable repertoire, despite the treatment occurring across both juvenile and adult life stages (Greene et al., 2018). There is also evidence that social conditions during the juvenile environment (tutor identity and number of males in tutoring group) affect syllable repertoire, but these were not tested during the breeding season (Gil et al., 2006). In line with the well‐known pattern of heightened neural plasticity in the song learning system during early development, that is, developmental plasticity (Table 2; Arnold, 1992; Larson, 2020), the juvenile developmental period is likely to be when this song trait is most environmentally sensitive.

## 4 DISCUSSION

In this review, we asked how timing, in addition to type of environmental variation, affects patterns of plasticity in sexual signal traits. As shown in our synthesis of traits in guppies, zebra finches, and house sparrows, applying a temporal perspective on trait plasticity can help to integrate and reconcile results of different studies. Without this perspective, these studies may seem to be in conflict with one another, with some providing evidence of trait plasticity and some finding no evidence of trait plasticity. Yet, in reality, traits may have differential sensitivity to their environment over time. In addition, these trait syntheses show how patterns of plasticity may differ among trait modalities, but we were unable to conduct a formal analysis due to the limitations of our dataset—namely, that most trait–environment associations were only tested in one life stage (e.g., all acoustic traits in amphibians were studied during the breeding season; Figure 4).

While researchers are increasingly conceptualizing sexual traits as plastic (Fox et al., 2019; Price, 2006; Ruell et al., 2013), and the theoretical importance of sensitive periods has been recognized in sexual selection (Hebets & Papaj, 2005; Ingleby et al., 2010; Møller & Pomiankowski, 1993), a key gap remains in designing, analyzing, and describing research on the temporal variation in phenotypic plasticity of sexual signal traits. Among the 123 papers that we scored in our review, information about life stage, or sexual maturity, of animal subjects was often not made explicit in favor of stating the absolute age of subjects or the calendar dates of manipulations. To make a determination about what life stage was studied, we sometimes had to cross‐reference with other papers in our review or make inferences based on other statements in the paper. The descriptions of experiments made it difficult or even impossible at times to assess the duration of an experimental treatment or the length of time between repeated measurements (but see Magris et al., 2018 for an excellent example of an experimental design diagram). In addition, the manipulations in some studies spanned multiple life stages, making it impossible to discern the onset and duration of a sensitive period in trait development. Research on plasticity of sexual signals has taken us a long way toward understanding the types of environmental influences that influence trait expression, but we are in need of more standardized reporting on the temporal aspect of environmental sensitivity of these traits (see Box 2 for our recommendations).

BOX 2 **Recommendations for future research**

Despite the well‐supported existence of sensitive periods in plastic traits, and the potentially important ways in which these sensitive periods could affect mate choice (Box 1) and divergence, our review uncovered several experimental design and reporting practices that obscure the importance of sensitive periods in sexual trait development. In order to better understand the temporal dimension of plasticity in sexually selected traits, we recommend that future researchers:

1. Justify their decision for studying a particular age or life stage of animal subjects. This should be done by considering the physiological mechanism(s) believed to underlie trait expression, and when those mechanisms are expected to be influenced by changes in environmental variables and induce change in trait expression.
2. Gather information about life stages separately. This can be done by (1) focusing on one life stage for a particular study, or (2) for longitudinal studies, by ensuring that repeated measurements are taken during the transition from one life stage to another, and for experimental studies, by swapping a subset of the control and treatment groups at the transition from one life stage to another. This allows researchers to discern the life stage during which a sensitive period occurs.
3. When using models of sexual selection as a framework for studying trait expression, evaluate the quality of evidence that the trait is linked with reproductive success. A trait linked with variables including territory quality and dominance, for example., is not necessarily sexually selected.
4. Include explicit information, such as a diagram of experimental design (e.g., Magris et al., 2018) showing the length of time between observations and/or treatments. All details of the experimental design and temporal nature of the study are critical for interpreting the results.
5. Limit inferences to the environmental variable and life stage under consideration. Trait plasticity at one time point does not imply trait plasticity (or lack thereof) at other time points.

Most traits in our dataset were only studied in one life stage across multiple studies. Presumably, researchers are selecting relevant life stages to study trait expression, but this is rarely made clear. Publication bias toward positive results could also explain the absence of papers studying environmental variation at life stages that do not influence trait expression. In addition, our dataset may have left out relevant papers. We are well aware that there is a rich and diverse set of literature that was not captured in our search, despite our efforts to use search terms that have been used in a variety of systems and over decades.

### 4.1 The temporal nature of plasticity and the information conveyed by sexual traits

The time at which trait expression is sensitive to the environment varies among traits. Some traits are plastic only during early development (developmental plasticity), some traits are plastic at particular, recurrent times (seasonal plasticity), and yet other traits remain plastic throughout life (continuous plasticity, Table 2; Arnold, 1992; Stamps, 2016). In reality, of course, most sexual traits are influenced by a mixture of inherited genetic information and environmental influences that are experienced over an animal's life, and thus, most traits will resist strict categorization. Nevertheless, identifying sensitive periods during which environmental exposure has the greatest impact on future trait expression is useful, as this provides important insights related to trait function and evolution (see Box 1).

Physiological knowledge of the trait in question is key to identifying likely periods of heightened plasticity. Many internal physiological mechanisms can mediate changes in trait expression following environmental changes, including oxidative stress (Hill, 2011), sex hormones (Bókony et al., 2008), stress hormones (San‐Jose & Fitze, 2013), inflammatory immune responses (Saino et al., 2013), insulin signaling (Warren et al., 2013), and more. Epigenetic changes, for example, DNA methylation, may result from changes in internal physiological status, particularly when there is a delay between the sensitive period and eventual trait expression (Laubach et al., 2018). The information conveyed by sexual traits is altered by the timing of plasticity of that trait (see Box 1), since expression of a plastic trait depends on not only what environmental influences the individual has experienced, but also when.

### 4.2 Implications for population divergence

Both the type and timing of environmental exposure may modify the information conveyed by sexual traits on an individual level, and selection on this variation at the individual level scales to affect population outcomes. Sexual selection has been viewed as potentially important in speciation processes (Panhuis et al., 2001; Ritchie, 2007; Safran et al., 2013; Servedio & Boughman, 2017; reviewed in Lindsay et al., 2019). Further, there is growing evidence that the environmental context in which populations exist is likely to have broad implications for the evolution of divergent sexual selection (Maan & Seehausen, 2011; Safran et al., 2013). For example, a recent study of closely related populations of barn swallows shows that different parasite populations are associated with the most divergent aspects of sexually selected traits (Hund et al., 2020), suggesting an interactive role of sexual selection and environmental context (parasites, in this case) in the differentiation of phenotype.

The role of plasticity is expected to modify the outcome of sexual selection (Cornwallis & Uller, 2010). Plasticity can dampen selection and thus maintain genetic diversity that is drawn from when populations are separated or begin to occupy new environments (Ingleby et al., 2010). Genotype‐by‐environment interactions in particular are increasingly studied in the context of sexual selection (Evans et al., 2015; Ingleby et al., 2010; Mills et al., 2007), an extremely valuable direction in helping to understand phenotypic variation and the divergence process. However, whether plasticity in sexual traits should accelerate or hinder divergence, and how population fitness may be affected, remains unclear (Fox et al., 2019). Key parameters likely include the degree to which environments differ, the degree of gene flow between populations (Ingleby et al., 2010), and sex differences in plasticity itself (Svensson et al., 2014). Despite increased interest in how plasticity affects sexual selection and divergence, the existence of sensitive periods in sexual traits is infrequently considered when studying how plasticity may modify evolutionary outcomes of sexual selection. Below, we consider how sensitive periods in plasticity of sexual traits may affect evolution and divergence, particularly in migratory or recently isolated populations.

#### 4.2.1 Developmental plasticity

Traits sensitive to early‐life conditions, such as syllable repertoire of zebra finches, can contribute to the divergence process. If preferences are formed by imprinting or traits are learned through tutoring, trait divergence can occur and be maintained even in the presence of gene flow (Fitzpatrick, 2012). If, however, signal information is lost or made unreliable due to environmentally sensitive period being separated in time from mate choice, selection may be reduced on preferences (Ingleby et al., 2010) leading to weaker sexual selection over time. In addition, changes in the developmental environment across generations can generate unreliable signals, in particular with strong genotype‐by‐environment interactions that exhibit environmental crossover (Mills et al., 2007).

#### 4.2.2 Seasonal plasticity

**FIGURE 6:**
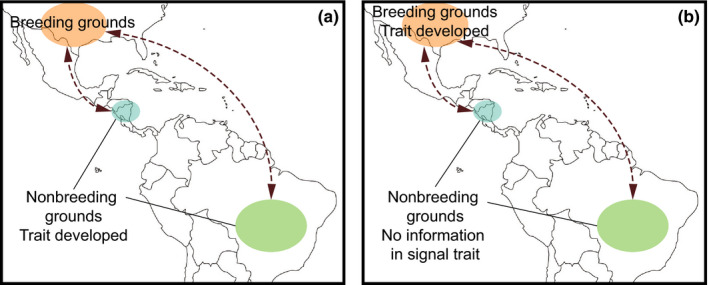
Conceptual diagram illustrating scenarios in which seasonal trait plasticity may (a) contribute to divergence or (b) erode divergence in migratory populations that share breeding locations but occupy different nonbreeding locations (i.e., there is a migratory divide). In (a), the trait is plastic during the time when the two populations are in two different nonbreeding locations; therefore, the environment could potentially differentially affect trait expression leading to phenotypic differentiation between the two populations upon return to their shared breeding grounds. In (b), the trait is plastic during the time when the two populations occupy the same breeding location; therefore, the environment cannot differentially affect trait expression among the two populations

For traits that are seasonally plastic, such as the black badge of house sparrows, analyses of environmental differences during the seasons when these traits are developed are most salient. An interesting case is the presence of migratory divides where individuals from overlapping breeding populations take different migratory routes to different wintering locations (Irwin, 2009; Turbek et al., 2018). If the traits used in mate selection are seasonally plastic and develop in environmentally different overwintering locations, trait differences may become coupled with nonbreeding location (Figure 6a). As such, when individuals arrive from different wintering locations they may return with increasingly different sexual traits that, when used in mate selection, may contribute to population divergence (Rolshausen et al., 2009). This is particularly true of “magic traits”—traits under divergent selection that also affect mate choice, such as breeding timing (Servedio et al., 2011). In contrast, if traits used in mate selection are seasonally plastic but develop in the shared breeding grounds, no environmentally relevant information about divergent migratory routes can be encoded in the sexual traits (Figure 6b) and divergence would not be expected to occur via sexual signaling.

#### 4.2.3 Continuous plasticity

For traits that are continuously plastic, such as the sigmoid display of guppies, the dynamics of trait expression and changes in immediate environmental context are targets for study. Even with the same trait and preference, movement to a novel environment (e.g., with novel parasites or new food sources) may affect the expression, cost, or honesty of trait, creating new selection on preferences (Maan & Seehausen, 2011) and potentially allow the evolution of new environment–trait linkages (Hund et al., 2020). While large‐scale environmental changes such as a warming climate are likely to affect all individuals in a region similarly, environmental variables such as parasites, predator density, and food resources can have patchy distributions and can differ between neighboring watersheds, ponds, valleys, or other kinds of geographical features that limit dispersal. These small‐scale environmental differences can affect trait expression and thus mate choice. Any movement of an individual between habitats would quickly induce changes in the migrant's trait expression, so lack of gene flow is the only conceivable way in which continuously plastic traits could contribute to population divergence.

## 5 CONCLUSION

In this review, we screened over 850 papers, which were then filtered for data collection on plasticity in studies of sexually selected traits in vertebrates. Our summary of this literature revealed that the existence of sensitive periods has been overlooked in studies of phenotypic plasticity of sexual traits. Further, a detailed synthesis of the most well‐studied traits (Table 3) shows that this perspective is critically important in enabling us to integrate results of different studies that have examined similar traits and environmental variables, but at different life stages. To highlight the importance of considering the timing of trait plasticity over an individual's lifetime, we (1) describe why phenotypic plasticity at different time points has important implications for understanding trait evolution within and between closely related populations, and (2) provide recommendations for future research.

Throughout, we outline patterns of plasticity that differ in *when* the environment affects trait expression. Although this temporal aspect of plasticity has been recognized for decades, there has been little empirical research on its implications for sexual selection. We advocate continued investigation into how plasticity shapes sexual selection, with particular attention to the timing of environmental interactions in altering animal physiology and expression of sexual traits. The timing of environment–trait interactions has important implications for trait evolution and population divergence.

## CONFLICT OF INTEREST

The authors declare that they have no conflicts of interest.

## AUTHOR CONTRIBUTIONS

**Molly T. McDermott:** Conceptualization (lead); Data curation (lead); Formal analysis (lead); Funding acquisition (equal); Methodology (equal); Resources (equal); Visualization (lead); Writing‐original draft (lead); Writing‐review & editing (lead). **Rebecca J. Safran:** Conceptualization (supporting); Data curation (supporting); Formal analysis (supporting); Funding acquisition (equal); Methodology (equal); Resources (equal); Writing‐original draft (supporting); Writing‐review & editing (supporting).

## Supporting information

Table S1‐S2

## Data Availability

All data used in this paper are from previously published and publicly available manuscripts listed in Table S1.
